# The COVID-19-designated hospitals in China: preparing for public health emergencies

**DOI:** 10.1080/22221751.2021.1931467

**Published:** 2021-06-03

**Authors:** Qian Li, Lin Wang, Bangfang Wang, Hongzhou Lu

**Affiliations:** a Department of Infectious Diseases, Shanghai Public Health Clinical Center, Shanghai, People’s Republic of China; b Department of Disease Control and Prevention, Shanghai Public Health Clinical Center, Shanghai, People’s Republic of China

**Keywords:** COVID-19, public health, nosocomial infection, centralized response and action system, emergencies

## Abstract

The designated hospitals are another health approach besides Fangcang shelter hospitals and newly built temporary hospitals for responding to COVID-19 epidemic in China. Faced with the emergency situation, about 1512 hospitals from 363 cities have been designated in China to tackle the spread of COVID-19. They were local hospitals repurposed by the Chinese government as a regional public health response. Their comprehensive services mainly include: “fever-clinics” to screen patients, COVID-19 department for higher-levels of medical care, and makeshift wards for emergencies. As the only COVID-19 designated hospital in Shanghai, we documented three characters (Centralized response and action system, Comprehensive functions, Closed-Loop Management System) and three strategies (Resource allocation, Prevention of nosocomial infection, Management during the post-COVID-19 pandemic stage) from the experience in responding to COVID-19 pandemic. Lastly, learning the lessons from COVID-19 pandemic, a more efficacy and rapid national response to public health emergencies is required. Serving as an essential component of public health system, the COVID-19-designated hospitals should be always prepared for future emergencies.

The designated hospitals are another health approach besides Fangcang shelter hospitals and newly built temporary hospitals for responding to the COVID-19 pandemic in China. Owing to limited resources in the makeshift hospitals, designated hospitals with more comprehensive functions, such as a more precise diagnosis and higher levels of medical care, are required to synergize with makeshift hospitals [1]. Most of the COVID-19 designated hospitals were regional infectious hospitals repurposed by the Chinese government for the rapid response to the COVID-19 pandemic.

As of February 2020, the COVID-19 outbreak in Wuhan reached its most serious situation, and three Fangcang shelter hospitals were temporarily constructed to contain overwhelming COVID-19 patients. In the time ensuing, more than 40 COVID-19 designated hospitals have been built, particularly for critical patients who require high-level medical care [2]. Shanghai, one of the world’s largest seaports and a major industrial and commercial centre in China was also faced with a high risk of being another epicentre in China due to its high population density. To avoid a shortage of medical resources for COVID-19 patients, Shanghai needed an approach to rapidly isolate and care for COVID-19 cases in a massive capacity.

Thus, it appointed Shanghai Public heath clinical center (SPHCC) as the only COVID-19 designated hospital in Shanghai City. SPHCC was constructed in 2004 after the spread of SARS in 2003 and has a long history of combating infectious diseases such as swine flu (H1N1), avian influenza A (H7N9), and Ebola virus disease (Ebola). The outbreak of COVID-19 was temporarily designated as the Shanghai regional response to COVID-19 based on the concept of centralized patients and rescue teams. In the past year, it has played a crucial role in controlling the spread of COVID-19 in Shanghai. By 17 April 2021, 1940 cases had been confirmed in Shanghai City (1874 recovered, 7 deaths, and 59 hospitalizations). Out of the 1940 cases, 1898 patients (97.84%) were treated in SPHCC, culminating in 1834 discharges (96.63%), 7 deaths (0.37%), and 57 hospitalizations (3.00%). Among the 1898 cases admitted to SPHCC, 381 were local cases (20.07%), 1515 were imported cases (79.82%), and 2 were import-related cases (0.11%). In addition, 1853 cases were mild (97.63%), 25 were moderate (1.05%), and 20 were severe (1.32%) (Table 1).

**Table 1.** COVID-19 patient flows in the Shanghai Public Health Clinical Center (SPHCC) from outbreak of the corona-virus disease 2019 to Apr 17, 2021.

Category	Hospitalizations			Discharges	Deaths	Total (%)
	Mild cases	Moderate cases	Severe cases			
Local cases	0	0	0	374	7	381 (20.07%)
Imported cases	56	0	1	1458	0	1515 (79.82%)
Import-related cases	0	0	0	2	0	2 (0.11%)
Total (%)	56 (2.95%)	0	1 (0.05%)	1834 (96.63%)	7 (0.37%)	1898 (100%)

The COVID-19 designated hospitals have three characteristics that make them contain the spread of COVID-19 effectively. The first characteristic is the centralized response and action system, which includes centralized patients, centralized experts, and centralized rescues [3]. The centralized working model has great advantages for improving human and medical resources during rapid rescue. The second characteristic is the massive capacity and comprehensive functions, including “fever-clinics” to screen patients, a COVID-19 department to isolate patients, and a large number of makeshift beds as emergency wards. At SPHCC, the COVID-19 department contains 527 beds in negative-pressure (NEP) rooms and more than 600 beds for high-level medical care, especially for those in critical conditions. In addition, 860 makeshift beds are made available for emergencies. The COVID-19 department also provides space for quarantining patients, essential living, social activities, and instruments such as extracorporeal membrane oxygenation (ECMO) for high-level medical care [4]. The third characteristic is a closed-loop management system. Measures taken for closed-loop management include reducing or shutting down patient transfer in the hospital, banning non-patient entry and relatives visitations, and isolating COVID-19 patients in the NEP rooms.

By sharing our previous experience with the COVID-19 designated hospital in Shanghai, three key strategies are summarized here. **First,** the hospital enhanced the efficiency of the COVID-19 response by centralizing resources. An Emergency Leadership Committee, which serves as the centre of management, was established (Figure 1). The committee consists of four teams: (1) an experts team, composed of qualified experts in the fields of emergency medicine, critical care medicine, infection prevention and control, respiratory medicine, etc.; (2) an infection prevention team, organized to prevent nosocomial infections through enhanced surveillance and evaluation, standardized clean and disinfection workflow, etc.; (3) a coordination team, set up to release real-time information through social media, including COVID-19 statistics, achievements, operational information, psychological comfort, scientific progress, etc.; and (4) a supporting team, aimed to support medical resource procurement, transport, stock, and accept social donations. In addition, goods and materials, including medical consumables, protective equipment, and scarce resources such as ECMO, were allocated according to the Chinese Guidelines for Diagnosis and Treatment of COVID-19 [5].

**Figure 1.**
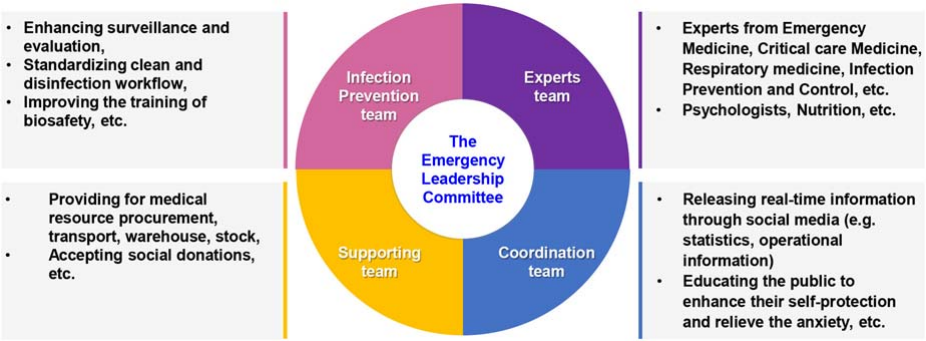
The human resource allocation strategies in response to COVID-19.

**Second,** achieving “zero nosocomial infection.” This is achieved in the hospital by formulating workflows to prevent nosocomial infections. The following measures were adopted: (1) Intensive education and training of healthcare providers on WHO recommendations [6]. (2) Formulate the discharge procedure for COVID-19 patients and healthcare providers about to leave COVID-19 wards and must be isolated for 1–2 weeks until they test negative in two RNA tests and undergo a body examination including a CT lung scan, according to the Chinese CDC guidelines [7]. (3) Enhancing environmental surveillance and disinfection in hospitals. The wards of discharged patients should be fully disinfected [8]. High-risk medical operations, such as treatment that produces droplets and aerosols, should be avoided or have their treatment time shortened if possible. If necessary, effective air isolation measures, including negative pressure rooms, frequent air-purifying respirators, and single-room allocation are required. After daily operations, all surfaces should be cleaned and disinfected in each department [9-12]. (4) Choosing proper clean and disinfection strategies according to different types of surfaces [13]. (5) Disposing of medical wastes correctly to reduce avoidable transmission according to the Chinese CDC guidelines [14]. For example, at the hospital, an incinerator is installed within a closed-loop system. The disposal procedure of medical wastes, such as transferring and incinerating, was recorded and tracked through an online system. In addition, the process of incinerating medical waste was automatic to avoid the risk of exposure to medical waste.

**Third,** it was well-prepared even at the post-COVID-19 pandemic stage. For example, (1) healthcare providers underwent regular COVID-19 screening to prevent hospital transmission. (2) Regular and professional trainings were conducted for all healthcare providers to promote preparedness and efficacy in crisis management. (3) The discharged COVID-19 patients were requested long-term follow-ups up to improve our understanding of the natural history of COVID-19 sequelae and evaluate the consequences of therapeutic interventions. (4) We advocating for vaccination through social media and accelerated equitable vaccination events.

Overall, COVID-19-designated hospitals were essential components of the public health system. Learning lessons from the COVID-19 pandemic, a more effective and rapid national response to public health emergencies is required. This needs future improvements in the COVID-19 designated hospitals. To this end, the following measures should be considered: (1) improving the digital information system to track the epidemic; (2) developing and applying medical robotics for high-risk operations during infection epidemics, such as clinical care (telemedicine and decontamination), logistics (delivery and handling of contaminated waste), diagnosis, and screening; and (3) strengthening scientific research to develop more precise and rapid diagnostic methods, more novel drugs, and efficacy vaccines for containing SARS-CoV2 and its variants. Finally, (4) to control the COVID-19 pandemic and prepare for future epidemics and disasters, the excellent work experience of the COVID-19 designated hospitals should be shared worldwide.
